# Treatment patterns and burden of behavioral disturbances in patients with dementia in the United States: a claims database analysis

**DOI:** 10.1186/s12883-019-1260-3

**Published:** 2019-02-28

**Authors:** Myrlene Sanon Aigbogun, Robert Stellhorn, Ann Hartry, Ross A. Baker, Howard Fillit

**Affiliations:** 1 Health Outcomes, Otsuka Pharmaceutical Development & Commercialization, Inc, 508 Carnegie Center, Princeton, New Jersey 08540 USA; 2 Health Economics and Outcomes Research, Lundbeck, LLC, Deerfield, IL USA; 3 Global Medical Affairs, Otsuka Pharmaceutical Development & Commercialization, Inc, Princeton, NJ USA; 4 Mount Sinai Medical Center, New York City, USA; 5 Alzheimer’s Drug Discovery Foundation, New York, NY USA

**Keywords:** Alzheimer’s disease, Dementia, Neuropsychological symptoms, Agitation, Behavioral disturbances, Healthcare resource utilization, Burden, Costs

## Abstract

**Background:**

Although patients with dementia frequently experience neuropsychological symptoms (NPS) such as agitation, which profoundly impacts patients, caregivers, and the healthcare system, few studies have evaluated the associated burden of agitation or agitation-related symptoms in dementia.

**Methods:**

This retrospective analysis of claims data from the Truven Health MarketScan® database (2012–2015) compared clinical characteristics, treatment patterns, healthcare resource utilization, and costs among patients with dementia with behavioral disturbances (BD) versus patients with dementia without BD. Existing BD diagnosis codes 294.11 or 294.21 were used as a means to identify patients with agitation/agitation-related symptoms.

**Results:**

From a starting sample of 6.4 million beneficiaries, 103,402 patients with dementia were identified, of whom 16,440 (16%) had BD during an average of 17 months of follow-up. Patients with BD had significantly more medical and psychiatric comorbidities and greater comedication use (i.e., antidementia drugs, antidepressants, and antipsychotics; all values, *P* < .0001) compared with patients without BD. A significantly greater number of hospitalizations, hospital days, outpatient hospital/clinic visits, number of skilled nursing visits, and number of patients with hospice visit were reported during follow-up in patients with BD compared with patients without BD (all values, *P* <  0.0001). Costs were also significantly higher among patients with BD versus those patients without BD ($42,284 vs. $32,640, respectively; *P* <  0.0001).

**Conclusions:**

Patients with dementia with BD had a higher prevalence of comorbidities, greater use of comedications, and greater healthcare utilization and costs than patients with dementia without BD.

**Electronic supplementary material:**

The online version of this article (10.1186/s12883-019-1260-3) contains supplementary material, which is available to authorized users.

## Background

Dementia, characterized by widespread progressive decline in cognitive and functional abilities and a wide range of challenging behavioral symptoms that occur throughout the disease process [1], is one of the major causes of disability and dependency among older people [2, 3]. Alzheimer’s disease (AD) is the most prevalent form of dementia, affecting approximately 5 million Americans in 2017 and accounting for 60–80% of dementia cases [2, 4].

Neuropsychiatric symptoms (NPS), a group of noncognitive symptoms and behaviors that occur in up to 90% of dementia patients over the course of their illness [5–7], present a challenging manifestation of dementia. Neuropsychiatric symptoms are grouped into four categories: mood disorders (e.g., depression, apathy, and euphoria), sleep disorders (e.g., insomnia, hypersomnia, and night-day reversal), psychotic symptoms (i.e., delusions and hallucinations), and agitation (e.g., pacing, wandering, sexual disinhibition, and aggression) [7]. It has been estimated that 40–60% of patients with AD exhibit symptoms of agitation and aggression [8].

Neuropsychiatric symptoms in dementia are associated with significant humanistic, societal, and economic burden [5–7, 9–12]. Agitation in dementia is associated with an increased rate of cognitive and functional decline [13], more rapid disease progression [14, 15], and earlier death [14] compared with dementia patients without agitation. Neuropsychiatric symptoms are associated with a reduced quality of life (QoL) for both the patient and the caregiver(s) [5, 10, 11, 16] and NPS may lead to caregiver burnout and a decrease in empathy [9]. Patients with dementia and NPS, including agitation, often require medication and are more likely to be admitted to an institution (i.e., care facility, general hospital inpatient, or mental health admission) and require early placement in long-term care than patients with dementia without NPS, contributing to increased overall costs of dementia care to the patient, caregivers, and the healthcare system [7, 9, 17–20].

The 2016 American Psychiatric Association (APA) Practice Guidelines provide recommendations for the treatment of agitation or psychosis in patients with dementia, suggesting a comprehensive, person-centered, non-pharmacological approach [21]. Antipsychotics are recommended within this approach as appropriate for the treatment of agitation and other NPS in patients with dementia, when symptoms are severe, are dangerous, and/or cause significant patient distress. However, at present, no medication has been approved by the United States (US) Food and Drug Administration (FDA) for the treatment of NPS associated with AD.

Although patients with dementia frequently experience NPS such as agitation, and NPS places a significant clinical and economic burden on patients, caregivers, and the healthcare system, few studies have evaluated the clinical and economic characteristics of agitation or agitation-related symptoms in dementia [5–7, 18, 22–25]. The objective of this retrospective claims study was to characterize the clinical and economic burden of BD in dementia by examining the clinical characteristics, treatment patterns, and healthcare utilization and costs associated with agitation in patients with dementia (including AD) using a large representative sample identified from the Truven MarketScan® Medicare 2012–2015 database. Because diagnosis codes were not available for agitation, BD diagnostic codes were used as a proxy to estimate the excess associated burden of agitation/agitation-related symptoms in dementia. While BD may not fully be representative of agitation, this was used as a proxy as they are the most appropriate diagnostic codes available.

## Methods

### Data source

Patients with dementia and BD were identified using medical and pharmacy claims data from the Truven Health MarketScan® Medicare Supplemental and Coordination of Benefits (COB) databases for 6.7 million Medicare beneficiaries for services provided from January 1, 2012, through September 30, 2015. The Truven Health MarketScan® database was selected because it is considered the gold standard in proprietary US healthcare databases, containing real-world data for healthcare research and analytics. The Truven Medicare database contains the healthcare experience of individuals with Medicare supplemental insurance paid for by employers. Both the Medicare Supplemental and COB provide detailed cost and utilization data from acute health care treatment, including claims for inpatient and outpatient (physician) care, laboratory and radiology services, emergency room (ER) use, and prescription drug fills, paid by Medicare or private insurance.

### Patient population

Selected patients had at least one International Classification of Diseases, Ninth Edition (ICD-9) or ICD-9 clinical modification (ICD-9-CM) code for Dementia/Dementia related (ICD-9: 290.0, 290.10, 290.11, 290.12, 290.13, 290.20, 290.21, 290.3, 290.40, 290.41, 290.42, 290.43, 294.0, 294.8, 294.10, 294.20; ICD-9-CM: 331.11, 331.2, 331.7) and AD (ICD-9-CM: 331.0) in any of four diagnosis fields on outpatient claims or any of five diagnosis fields on inpatients claims. Of these, 398,128 patients were at least 65 years old, and 103,402 were continuously enrolled in the Medicare supplemental database and had continuous health plan enrollment with medical and pharmacy benefits for at least 6 months pre-index period (baseline) and 6 months post-index date. Existing BD diagnostic codes 294.11 and 294.21 were used as a proxy for agitation because ICD-9 diagnostic codes were not available to identify agitation. To identify patients with late-stage disease, patients were flagged with late-stage disease per Fillit et al. 2002 criteria [26]: presence of decubiti (707.00), malnutrition (260, 261, 262, 263.1, 263.2, 263.8, 263.9), and aspiration pneumonia (507.x).

### Study measures and analyses

Descriptive comparative analyses were conducted for patients with dementia/AD with BD versus patients without BD. Additional analyses were conducted excluding patients with late-stage disease. For significance testing, continuous variables were analyzed using Student’s *t*-tests and categorical variables were analyzed using Chi-square tests. An alpha level of 0.05 was used for all tests. All analyses were conducted using SAS Enterprise Guide 7.1 (SAS Institute Inc., Cary, NC).

### Baseline patient demographics

Age (grouped by 65–74, 75–84, 85–94, and > 95 years), gender, geographic region (northeast, north central, south, west, and unknown), Charlson Comorbidity Index (CCI) score (0, 1, 2, 3, or 4+), and follow-up length were examined. For continuous study measures, the mean, standard deviation or 95% confidence interval (CI), median, and interquartile range were calculated. For categorical measures, the number and percentage of patients were calculated.

### Common medical comorbidities at baseline

At baseline, common medical comorbidities among patients with and without BD were analyzed. See Additional file 1 TableS1 for a list of the conditions that were included, and their respective ICD-9-CM diagnosis codes.

### Common concomitant medications at baseline

Prescription claims during the follow-up period were identified for the following concomitant medication categories: antipsychotics, antidepressants, hypnotics, anticoagulants, antiplatelets, nonsteroidal anti-inflammatory drugs (NSAIDs), antihistamines, anticholinergics, antidiabetics, antihypertensives, anti-epileptics, anti-emetic neuroleptics, antidementia drugs, narcotics, hormones, stimulants, for dyslipidemia, and for Parkinson’s disease. See Additional file 2 TableS2 for a list of the medications that were included under each category.

### Antipsychotic treatment patterns

The number and percentage of patients for each index antipsychotic treatment, after dementia/AD diagnosis was captured. Additional characteristics were identified, including: time to index treatment from dementia/AD diagnosis date; time to index treatment from date of BD diagnosis date; medication possession ratio (MPR) for antipsychotics and common concomitant medications (i.e., total days of supply of index treatment over 12-month and entire follow-up); the number of antipsychotic prescriptions and the number of different antipsychotics prescribed during the observation period; and the mean time to antipsychotic discontinuation. Patients were considered discontinued if there was no additional refill for the index treatment component 45 days after the run-out date of the previous prescription, or if the patient was prescribed a new drug without any subsequent prescription for the index drug component; for patients with index monotherapy, patients were considered discontinued if the patient was prescribed a new drug with ≥14-day overlap with the index drug. The discontinuation date was considered the run-out date of the previous prescription, or the occurrence of the new drug, according to discontinuation. Among the two most commonly used antipsychotics, the doses of the initial prescription and the highest dose of all prescriptions were also captured.

### Healthcare resource utilization

The following measures of healthcare utilization were examined: number of hospitalizations per-patient per-year (PPPY), number of hospital days PPPY, number of outpatient hospital/clinic visits PPPY, number of physician visits PPPY, number of skilled nursing visits, and number of patients with hospice visit.

### Healthcare costs

All-cause total, inpatient, outpatient, ambulatory, and pharmacy costs were calculated PPPY. All-cause total healthcare costs were also stratified by number of comorbidities, according to CCI score (CCI 0, 1, 2, and 3).

## Results

### Baseline patient demographics

**Fig. 1:**
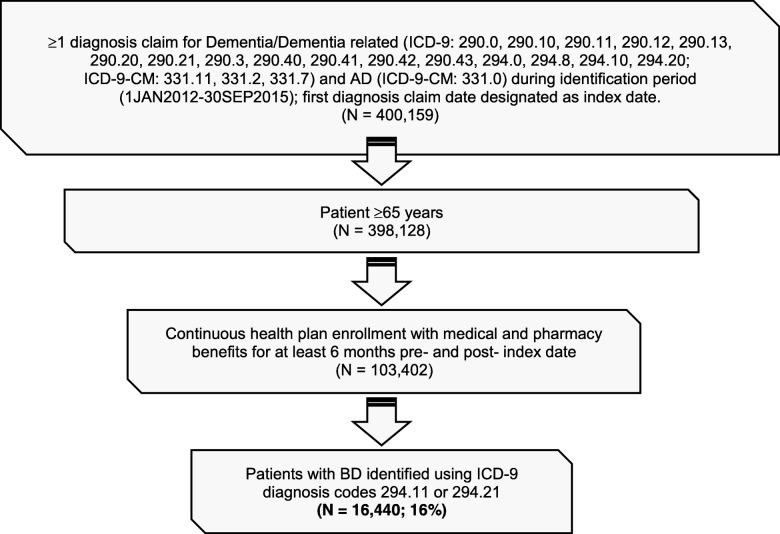
Study sample selection for patients with dementia with and without BD. Legend: *BD* behavioral disturbance, *CM* clinical modification, *ICD* International Classification of Diseases

From a starting sample of 6.4 million beneficiaries, 103,402 eligible patients with dementia/AD met the study inclusion criteria. Behavioral disturbances were identified in 16% (*n* = 16,440) of patients with dementia/AD (Fig. 1). Fifty-three percent (*N* = 8749) of patients identified with BD had their initial BD diagnosis claim occurring within a year after the index date during the follow-up period. Baseline patient demographics of patients with and without BD are presented in Table 1. The mean age of both groups was approximately 83 years. A significantly greater proportion of patients with BD were 85–94 years compared with patients without BD (43.0% vs 40.0%, respectively; *P* <  0.0001). Twenty percent of patients with BD were categorized as late-stage (per the Fillit 2002 late stage criteria) versus 13% of patients without BD group. The length of follow-up was approximately 17 months.

**Table 1:** Baseline patient demographics among patients with and without BD

	With BD (*N* = 16,440)	Without BD (*N* = 86,962)	*P* value (with versus without BD)
Mean age (years [SD])	83.5 (7.1)	82.9 (7.5)	< 0.001
Age group (*n* [%])			
65–74	1981 (12.1)	13,247 (15.2)	< 0.001
75–84	6628 (40.3)	34,824 (40.1)	
85–94	7066 (43.0)	34,811 (40.0)	
95+	765 (4.7)	4080 (4.7)	
Gender (n [%])			
Male	6478 (39.4)	33,691 (38.7)	0.110
Female	9962 (60.6)	53,271 (61.3)	
Geographic region (n [%])			
Northeast	3222 (19.6)	17,724 (20.4)	< 0.001
North	6290 (38.3)	31,530 (36.3)	
South	4868 (29.6)	25,635 (29.5)	
West	2020 (12.3)	11,809 (13.6)	
Unknown	40 (.2)	264 (.3)	
Mean follow-up (months [SD])	16.99 (7.1)	15.80 (7.0)	< 0.001

*BD* behavioral disturbance, *SD* standard deviation

### Common medical comorbidities at baseline

Patients with BD had a greater prevalence of medical and psychiatric comorbidities at baseline compared with patients without BD. Considering 53% of the patients in the BD group had their initial BD diagnosis claim within the year after the index date, we also assessed prevalence rates of comorbidities among those with 12 months of follow up data. Table 2 presents common medical and psychiatric comorbidities in patients with and without BD at baseline and at selected follow-up (12 months post-index). A significantly greater number of patients with BD than patients without BD had the following psychiatric comorbidities: altered mental status (16.9% vs 11.4%), anxiety disorder (9.1% vs 7.2%), depression (16.0% vs 12.4%), mood disorder (16.7% vs 13.3%), psychotic disorder (19.0% vs 12.0%), and psychosis (16.1% vs 10.1%) (all values, *P* <  0.0001).

**Table 2:** Common medical comorbidities at baseline and selected follow-up in patients with and without BD

	Baseline (6-months pre-index date)			Selected Follow-up (12 months post-index)		
	With BD (N = 16,440)	Without BD (N = 86,962)	*P* Value	With BD (*N* = 11,440)	Without BD (*N* = 54,516)	*P* Value
Medical Comorbidities (%)						
Hypertension	55.3%	58.0%	< 0.001	80.6%	77.7%	< 0.001
Dyslipidemia	23.4%	26.7%	< 0.001	41.2%	42.8%	0.001
Diabetes	24.1%	25.5%	< 0.001	30.8%	30.3%	0.332
Cerebrovascular disease	20.2%	21.0%	0.019	39.7%	34.5%	< 0.001
Urinary tract infection	17.8%	15.4%	< 0.001	39.6%	27.2%	< 0.001
Chronic Pulmonary Disease	14.4%	16.3%	< 0.001	25.1%	24.1%	0.021
Congestive Heart Failure	12.9%	13.7%	0.004	23.7%	20.9%	< 0.001
Peripheral vascular disease	8.4%	8.9%	0.068	18.4%	15.6%	< 0.001
Hip fracture and other fractures	10.1%	9.5%	0.024	21.1%	16.0%	< 0.001
Malignancy	8.0%	9.8%	< 0.001	11.6%	13.3%	< 0.001
Malnutrition	2.4%	2.0%	0.0004	6.1%	3.8%	< 0.001
Pneumonia	6.8%	7.1%	0.231	17.4%	13.3%	< 0.001
Psychiatric Conditions (%)						
Mood disorder	16.7%	13.3%	< 0.001	41.6%	25.7%	< 0.001
Depression	16.0%	12.4%	< 0.001	40.4%	24.4%	< 0.001
Delirium	6.95%	4.57%	< 0.001	21.0%	9.5%	< 0.001
Psychotic disorders	19.0%	12.0%	< 0.001	46.7%	21.4%	< 0.001
Psychosis	16.1%	10.1%	< 0.001	39.7%	17.8%	< 0.001
Altered Mental State	16.9%	11.4%	< 0.001	43.1%	21.6%	< 0.001
Anxiety Disorder	9.1%	7.2%	< 0.001	26.1%	14.6%	< 0.001

*BD* behavioral disturbance

### Common concomitant medications at baseline

**Fig. 2:**
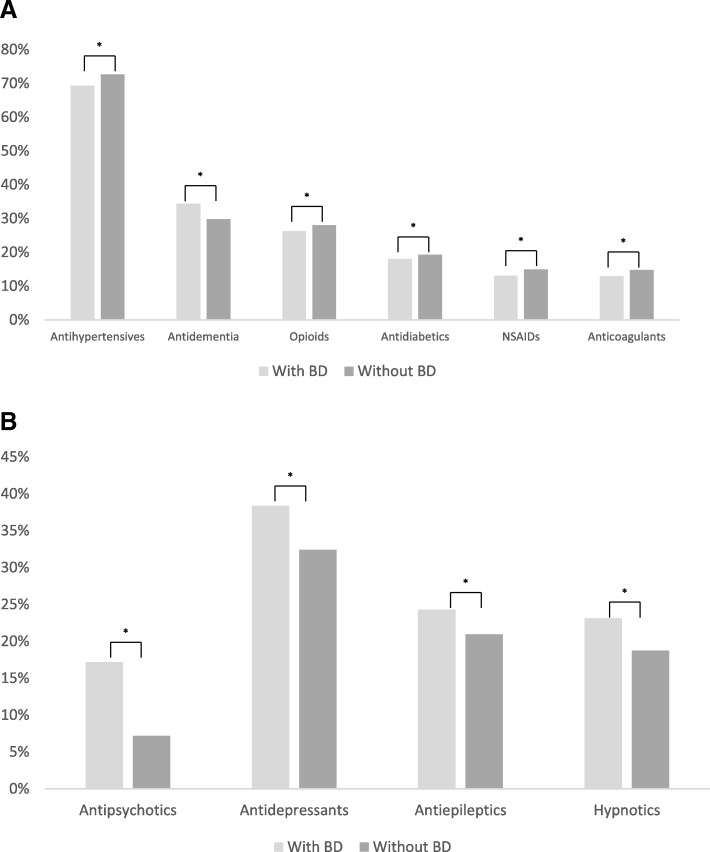
Common concomitant medications at baseline for patients with and without BD (**a**) Somatic medications (**b**) Psychiatric medications. Legend: *P < .0001, *BD* behavioral disturbance, *NSAID* non-steroidal anti-inflammatory drug

Figure 2 displays common concomitant medications at baseline, grouped by somatic (i.e., antihypertensives, antidementia, antidiabetics, NSAIDs, anticoagulants, and opioids) and psychiatric (i.e., antipsychotics, antidepressants, antileptics, hypnotics) medications. A significantly greater proportion of patients with BD than patients without BD, respectively, were treated with antidementia drugs (34.4% vs 29.8%), antidepressants (38.4% vs 32.4%), antipsychotics (17.2% vs 7.2%), and hypnotics (23.1% vs 18.7%) (all values, *P* <  0.0001). Among antidementia treatment, a significantly greater number of BD patients compared with patients without BD, respectively, were treated with memantine (15.5% vs 11.6%; *P* <  0.0001), donepezil (23.5% vs 21.3%; *P* <  0.0001), rivastigmine (5.4% vs 4.0%; *P* <  0.0001), and galantamine (1.1% vs .9%; *P* = 0.0027).

### Psychotropic treatment patterns

The most common psychiatric drugs among both populations at baseline were antidepressants, anti-convulsants, and hypnotics, all of which were used at somewhat higher rates among patients with BD than in the patients without BD. In contrast, antipsychotics were used the least in both populations, but were used substantially more among patients with BD compared with patients without BD. During the entire observation period, the proportion of patients with BD on antipsychotic treatment was significantly greater (54% vs 16%, *P* <  0.001). At baseline, a significantly greater proportion of BD patients than patients without BD were treated with the antipsychotics quetiapine (24% vs 7%) and risperidone (13% vs 3%) (both values, *P* <  0.0001). Table 3 provides a breakdown of the initial dose and highest dose of quetiapine and risperidone according to dosage strength among patients with BD. Dosage strength was increased from the index dose to the highest dose for both quetiapine and risperidone.

**Table 3:** Initial and highest dose of quetiapine and risperidone: A) patients with BD, B) patients without BD

A)					
Quetiapine (*N* = 3927)			Risperidone (*N* = 2108)		
Strength	Initial Dose (n, %)	Highest Dose (n, %)	Strength	Initial Dose (n, %)	Highest Dose (n, %)
25 mg	2904 (74.0%)	2050 (52.2%)	< .5 mg	978 (46.4%)	648 (30.7%)
50 mg	726 (18.5%)	1155 (29.4%)	.5 mg	762 (36.2%)	842 (39.9%)
100 mg	192 (4.9%)	497 (12.7%)	.6–.9 mg	0	0
150 mg	16 (.4%)	28 (.7%)	1 mg	302 (14.3%)	492 (23.3%)
200 mg	58 (1.5%)	97 (2.5%)	1.1–1.9 mg	0	0
300 mg	19 (.5%)	75 (1.9%)	2 mg	43 (2.0%)	79 (3.8%)
400 mg	12 (.3%)	25 (.6%)	> 2 mg	23 (1.1%)	47 (2.2%)
B)					
Quetiapine (*N* = 5802)			Risperidone (*N* = 2835)		
Strength	Initial Dose (*n*, %)	Highest Dose (*n*, %)	Strength	Initial Dose (*n*, %)	Highest Dose (*n*, %)
25 mg	4218 (72.7%)	3509 (60.5%)	< .5 mg	1209 (42.6%)	928 (32.7%)
50 mg	1041 (17.9%)	1422 (24.5%)	.5 mg	1054 (37.2%)	1117(39.4%)
100 mg	314 (5.4%)	526 (9.1%)	.6–.9 mg	0 (0%)	0
150 mg	34 (.6%)	56 (1.0%)	1 mg	429 (15.1%)	591 (20.9%)
200 mg	98 (1.7%)	149(2.6%)	1.1–1.9 mg	0	0
300 mg	72 (1.2%)	94(1.6%)	2 mg	83 (2.9%)	107 (3.8%)
400 mg	25 (.4%)	46 (.8%)	> 2 mg	60 (2.1%)	92 (3.2%)

Twenty-three percent of patients with BD with index antipsychotic treatment were switched to another antipsychotic treatment during follow-up versus 11% of patients without BD; the mean time to switch index antipsychotic treatment to another antipsychotic treatment was 107.2 ± 137.0 days in patients with BD versus 114.1 ± 141.1 days for patients without BD. The medication possession ratio was similar among patients with BD (.72 ± 1.43) versus patients without BD (.74 ± 1.27). In both groups, a majority of patients received only one type of antipsychotic treatment during the observation period (with BD = 70% versus without BD = 85%). Twenty-two percent of patients with BD had two different AP treatments versus 13% of patients without BD, and 6% of patients with BD had three different AP treatments versus 2% of patients without BD.

### Healthcare resource utilization

A greater number of hospitalizations (mean number PPPY [95% CI]: with BD = 0.97 [0.96–0.99] vs without BD = 0.62 [0.62–0.63], *P* <  0.001), hospital days (mean number PPPY [95% CI]: with BD = 6.80 [6.61–6.99] vs without BD = 3.55 [3.48–3.61], *P* <  0.001), outpatient hospital/clinic visits (mean number PPPY [95% CI]: with BD = 6.41 [6.28–6.55] vs without BD = 5.79 [5.73–5.84], *P* <  0.001), number of skilled nursing visits (mean number PPPY [95% CI]: with BD = 2.25 [2.20–2.29] vs without BD = 1.13 [1.12–1.15], *P* <  0.001), and number of patients with hospice visit [percentage of patients, 95% CI]: with BD: 321 [1.95, 1.75–2.18%] vs without BD = 798 [0.92, 0.86–0.98%], *P* <  0.001) were reported during follow-up in patients with BD compared with patients without BD. However, the number of physician visits was higher during follow-up for patients without BD compared with patients with BD (with BD = 8.53 (8.38–8.68) vs without BD = 11.69 (11.60–11.77), *P* <  0.001).

### Healthcare costs

During follow-up, mean PPPY total healthcare costs (95% CI) were significantly higher in patients with dementia/AD with BD ($42,284 [$41,383–$43,184) versus without BD ($32,640 [$32,246–$33,034]; *P* <  0.0001) (see Table 4). Inpatient, outpatient ER, and ambulatory costs were significantly higher in patients with BD compared to those without BD; however, pharmacy costs were significantly higher in the without BD group versus the with BD group (see Table 4; all values, *P* <  0.0001). To evaluate whether treatment adherence may have impacted pharmacy costs between groups, MPRs were calculated for the most commonly used comedications. In patients with follow-up data for 12 months, the mean MPRs were lower for those with BD compared to patients without BD for antihypertensives (0.72 [0.71–0.73] vs 0.82 [0.81–0.83]), antidiabetics (0.78 [0.75–.81] vs 0.87 [0.86–0.89]), antidepressants (0.75 [0.74–0.77] vs 0.83 [0.81–0.84]), and antidementia (0.74 [0.73–0.76] vs 0.83 [0.81–0.84]) medications.

**Table 4:** Healthcare costs during follow-up, per-patient-per-year

	With BD (95%CI)	Without BD (95%CI)	Difference	*P* value (with versus without BD)
Total Costs	$42,284 ($41,383–$43,184)	$32,640 ($32,246–$33,034)	$9644	< 0.0001
Inpatient Costs	$17,781 ($17,131–$18,431)	$12,374 ($12,122–$12,627)	$5407	< 0.0001
Ambulatory Costs	$17,918 ($17,476–$18,361)	$14,072 ($13,849–$14,295)	$3846	< 0.0001
Pharmacy Costs	$4105 ($4011–$4200)	$4447 ($4384–$4511)	-$342	< 0.0001
Outpatient ER Costs	$2479 ($2380–$2579)	$1746 ($1711–$1781)	$733	< 0.0001

*BD* behavioral disturbance, ER emergency room

Total healthcare costs (95% CI) stratified by the number of CCI comorbidities demonstrated significantly higher costs among the with BD group compared with the without BD group for patients with a CCI score of 0 ($33,382 [$32,398–$34,367] vs $20,886 [$20,503–$21,270], respectively), CCI score of 1 ($37,588 [$36,166–$39,010] vs $27,705 [$27,125–$28,285], respectively), CCI score of 2 ($45,465 [$42,927–48,003] vs $32,980 [$32,149–$33,812]), and CCI score of 3 ($51,449 [$47,557–$55,341] vs $41,398 [$40,005–$42,793]) (all values, *P* <  0.0001).

## Discussion

This retrospective analysis identified novel information regarding the clinical characteristics, treatment patterns, and healthcare utilization and costs of patients identified as having dementia/AD with BD using one of the largest comprehensive patient samples available, providing insight into an underrepresented area of research.

Although NPS are, at some point in their illness, exhibited in almost all people with dementia, this study identified significant baseline differences in patient demographics between those with and those without BD. Patients with dementia with BD were older among the 85–94 subgroup and had greater disease comorbidity than patients without BD. They also had a higher prevalence of medical and psychiatric conditions, notably, mood disorders, psychotic disorders, and altered mental status. Of note, rates of hip fractures and inflammatory conditions such as pneumonia, and urinary tract infections were also greater in patients with BD. This may suggest that in this population, BDs may be primary due to their AD/Dementia condition or could be secondary due to infections or major surgery.

Baseline treatment patterns were also significantly different among patients with and without BD. Patients with BD had significantly greater use of comedications, including antidementia medications, antipsychotics (e.g., quetiapine and risperidone), and antidepressants, compared with patients without BD. It is notable that quetiapine and risperidone doses were lower than those recommended for treatment of schizophrenia/psychosis, though we cannot determine from the data whether lower doses were chosen due to the elderly status of patients or other psychiatric co-medications, or other reasons. At present, no pharmacological treatment has been FDA-approved for the treatment of agitation in dementia in the US [21]. Antipsychotics and antidepressants are typically used off-label to treat NPS in dementia [27]. The 2016 APA Practice Guidelines currently only recommend the use of antipsychotics for the treatment of agitation and other NPS in patients with dementia when symptoms are severe, are dangerous, and/or cause significant patient distress [21]. Antipsychotics are associated with significant side effects and an increased risk of stroke and death in older adults with psychosis and dementia; therefore, the decision to use an antipsychotic drug should be considered with caution [27, 28]. The higher use of antipsychotics and antidepressants among the dementia patients with BD compared with patients without BD, in the current study, highlights potential off-label use of these medications for BD, underscoring the unmet need for a therapy with demonstrated effectiveness to treat BD in dementia.

Polypharmacy in patients with dementia is typically due to the significant comorbidity of medical and psychiatric conditions [29]. According to Desai and colleagues, polypharmacy increases the possibility of a “prescribing cascade”, in which side effects of drugs are misdiagnosed as symptoms of another medical condition resulting in further prescriptions and side effects [9]. Polypharmacy is also associated with a high incidence of drug-drug reactions and may manifest as BD [9]. Few studies have examined the association between polypharmacy and dementia [30, 31] and these studies did not evaluate how polypharmacy or multimorbidity might influence behavioral symptoms in patients with dementia. Therefore, further research is needed to elucidate how polypharmacy and comorbidities affect BD in patients with dementia.

Given the risks of polypharmacy for older patients, particularly those with dementia [32], numerous behavioral care strategies have been studied as potential treatment for BD in dementia. Recent research suggests that use of behavioral care strategies that focus on preserved capabilities and interests in the patient with dementia and engagement of caregivers as promising approaches for reducing NPS in dementia [33]. These activities are thought to engage patients in a positive manner, potentially minimizing agitation or other NPS. In addition, instructions to caregivers to involve patients with dementia in activities may minimize their time spent caregiving and may enhance their own well-being [33].

American, British and Canadian guidelines recommend alternative clinical approaches as a first-line intervention including ruling out underlying causes and initiating and attempting non-pharmacological interventions such as behavioral interventions [34]. The most recently 2016 APA Practice Guidelines recommended a comprehensive, person-centered non-pharmacological approach prior to initiating the use of pharmacological treatment [21]. Although a range of behavioral techniques are available, notably they have a limited evidence base. Because behavioral care strategies that include the engagement of caregivers have been shown to potentially minimize agitation or other NPS [33], it would also be of interest to evaluate whether patients with dementia and BD have more limited access to caregivers in future analyses.

Few studies have examined the economic burden of agitation in dementia. In a recent systematic literature review of the economic burden of agitation in AD, only four studies were identified [18]. The findings of the systematic literature review suggest an increase in economic burden associated with agitation; however, the majority of the identified estimates on the economic burden were derived outside the US [22, 23, 25], and only one study was US-based [24]. In the current study, healthcare resource utilization and costs were higher among patients with dementia and BD compared with patients without BD. Hospitalizations, outpatient hospital/clinic visits, and the number of skilled nursing days were significantly higher for patients with BD versus patients without BD. The difference in total healthcare costs among patients with and without BD was approximately $10,000 PPPY (30% higher); and inpatient, outpatient ER, and ambulatory costs were significantly higher in patients with BD compared with patients without BD. In contrast, patients with BD had lower utilization for physician office visits, which may be indicative of a failure of ambulatory care as a contributing factor to hospitalizations. Lower costs for prescription drugs in patients with BD compared to patients without BD may be due to lower rates of adherence, as well as caregiver burden regarding behaviors, and less time available for managing prescriptions. The pattern of higher ambulatory costs and lower costs for physician visits and prescription drugs has been observed previously in a cohort of dementia patients in a database analysis by Hill and colleagues [35]. The high medical burden of patients with BD and AD is an opportunity for the medical community to partner with caregivers to ease the burden of care. Similar analyses in long-term care populations would also be of interest.

Using claims data to investigate research has inherent limitations. In the current study, claims data included only 3 years (2012 to 2015), which might not reflect holistic characteristics and treatment patterns of patients with dementia who have BD. Identification of agitation was limited to available ICD-9 diagnostic codes for BD (294.11 or 294.21) because ICD-9 diagnostic codes are not available to identify agitation; therefore, the prevalence of diagnosis might be underestimated. In addition, treatment was not examined as a marker for BD. Additional proxy markers may be considered to identify patients with agitation-related symptoms. For example, during the observation period, 7 and 15% of patients in the overall sample initiate antipsychotic treatment without a psychosis or depression diagnoses, respectively. Moreover, while we have assessed a proxy for late-stage disease, identifying or adjusting for severity of AD or dementia in analyses using administrative claims data is not possible because billing codes for AD do not directly indicate severity, and cognitive tests such as Mini-Mental State Examination (MMSE) or Montreal Cognitive Assessment (MOCA) are not included. Furthermore, the occurrence of coding errors or diagnoses entered for administrative processing rather than clinical completeness may limit accuracy of data.

Different neurodegenerative diseases may be associated with certain NPS; however, few studies have examined differences in NPS between dementia subtypes and the results of such studies have been equivocal [36–39], making it difficult to establish a pattern of NPS in different subtypes of dementia. In the current study, analyses were conducted in patients with all-cause dementia. Further analyses are needed to determine if distinct patterns would emerge by dementia subtypes.

Although it has been estimated that up to 90% of patients with dementia experience NPS over the course of their illness [5–7], the rate of BD in patients with dementia in the current claims analysis is low (16%). Lower rates observed in this study potentially indicate underreporting of BD, under-coding of BD, or lack of symptom recognition and treatment. Patients with more contacts to healthcare professionals may have a higher probability that their BD is recorded with an ICD-9 diagnosis code than patients with fewer visits to healthcare professionals. This disparity in the prevalence rates of BD in dementia in the current study highlights the need for additional research to understand the diagnostic pathway to BD in dementia.

## Conclusions

In this US claims database analysis, patients with dementia and BD had significantly more medical and psychiatric disease comorbidities and were treated more frequently with antidementia drugs, antidepressants, antipsychotics, and antileptics, compared with patients without BD. Patients with dementia and BD had substantially greater healthcare resource utilization (i.e., hospitalizations, hospital days, outpatient hospital/clinic visits, and number of skilled nursing visits), and as a result, incurred significantly greater costs, compared with patients without BD, highlighting the significant economic burden of BD in dementia among the US Medicare population.

## Additional files

Additional file 1: **TableS1.** Individual medical comorbidities collected for patients with and without BD. This table lists all of the ICD-9-CM diagnostic codes for the comorbid medical disorders observed in the study population. (DOCX 13 kb)

Additional file 2: **TableS2.** All medications including antipsychotics and anti-dementia treatments collected for patients with and without BD. This table lists all of the medications taken by patients in the study, including antipsychotics and anti-dementia therapeutics. (DOCX 17 kb)

## Abbreviations

AD: Alzheimer’s disease
APA: American Psychiatric Association
BD: Behavioral disturbances
CCI: Charlson Comorbidity Index
CI: Confidence interval
COB: Coordination of Benefits
ER: Emergency room
FDA: Food and Drug Administration
ICD-9 CM: International Classification of Diseases, ninth edition, clinical modification
ICD-9: International Classification of Diseases, ninth edition
MMSE: Mini-Mental State Examination
MOCA: Montreal Cognitive Assessment
MPR: Medication possession ratio
NPS: Neuropsychological symptoms
NSAID: Nonsteroidal anti-inflammatory drug
PPPY: Per-patient per-year
QoL: Quality of life
US: United States
