# Supplementary material for: Treatment patterns and burden of behavioral disturbances in patients with dementia in the United States: a claims database analysis
Source: BMC Neurol. 2019 Feb 28;19:33. doi: 10.1186/s12883-019-1260-3 (PMC6396493; doi:10.1186/s12883-019-1260-3)
Supplement: Supplementary file 1 — TableS1. Individual medical comorbidities collected for patients with and without BD. This table lists all of the ICD-9-CM diagnostic codes for the comorbid medical disorders observed in the study population. (DOCX 13 kb) [file 12883_2019_1260_MOESM1_ESM.docx]

| **Condition** | **ICD-9-CM Diagnosis Code** |
| --- | --- |
| Congestive Heart Failure | 428 |
| Dementia | 290 |
| Chronic Pulmonary Disease | 490-496, 500-505, 506.4 |
| Rheumatologic disease | 710.0, 710.1, 710.4, 714.0-714.2, 714.81, 725 |
| Peptic Ulcer Disease | 531-534 |
| Moderate or Severe Liver Disease | 572.2-572.8, 456.0-456.21 |
| Diabetes with chronic complications | 250-250.3, 250.4-250.6, 250.7 |
| Hemiplegia or Paraplegia | 344.1, 342 |
| Renal Disease | 582, 583-583.7, 585, 586, 588 |
| Any malignancy, including leukemia, lymphoma | 140-172, 174-195.8, 200-208 |
| Myocardial Infarction | 410, 412 |
| Peripheral Vascular Disease | 443.9, 441, 785.4, V43.4, procedure code 38.48 |
| Cerebrovascular Disease | 430-438 |
| Mild Liver Disease | 571.2, 571.5, 571.6, 571.4 |
| Diabetes with chronic complications | 250.4-250.6 |
| Metastatic solid tumor | 196-199.1 |
| AIDS | 042, 043, 044 |
| Parkinson’s Disease | 332 |
| Epilepsy | 345.xx, 780.39 |
| Mood Disorder  (including major depression, bipolar affective disorder, and depression not elsewhere classified) | 296.xx, 300.4, 311 |
| Pneumonia | 486 |
| Pain | 780.96, 338.xx |
| Psychosis | 295.xx-299.xx |
| Schizophrenia | 295.xx |
| Other psychosis | 296.xx-299.xx |
| Hip Fractures | 821 and 959 |
