# Supplementary material for: Treatment patterns and burden of behavioral disturbances in patients with dementia in the United States: a claims database analysis
Source: BMC Neurol. 2019 Feb 28;19:33. doi: 10.1186/s12883-019-1260-3 (PMC6396493; doi:10.1186/s12883-019-1260-3)
Supplement: Supplementary file 2 — TableS2. All medications including antipsychotics and anti-dementia treatments collected for patients with and without BD. This table lists all of the medications taken by patients in the study, including antipsychotics and anti-dementia therapeutics. (DOCX 17 kb) [file 12883_2019_1260_MOESM2_ESM.docx]

| **Class** | **Drug Name** |
| --- | --- |
| **Antipsychotics** | aripiprazole, asenapine, brexpiprazole, cariprazine, chlorpromazine, clozapine, fluphenazine, haloperidol, iloperidone, loxapine, lurasidone, mesoridazine, molindone, olanzapine, paliperidone, perphenazine, perphenazine/amitriptyline, pimavanserin, pimozide, prochlorperazine, promazine, quetiapine, risperidone, thioridazine, thiothixene, trifluoperazine, ziprasidone |
| **Antidepressants** | amitriptyline, amoxapine, bupropion, citalopram, clomipramine, desipramine, desvenlafaxine, doxepin, duloxepine, escitalopram, fluoxetine, fluvoxamine, imipramine, isocarboxazid, maprotiline, mirtazapine, nefazodone, nortriptyline, paroxetine, phenelizine, protriptyline, selegiline, sertraline, tranylcypromine, trazodone, trimipramine, venlafaxine, vilazodone |
| **Hypnotics** | **Benzodiazepines:** flurazepam, clonazepam, quazepam, triazolam, lorazepam, alprazolam, temazepam, oxazepam, prazepam, estazolam, flunitrazepam, chlordiazepoxide, clorazepate, diazepam, midazolam  **Non-Benzodiazepines:** eszopiclone, zaleplon, zolpidem, ramelteon  **Barbiturates:** allobarbital, amobarbital, aprobarbital, alphenal, barbital, brallobarbital, pentobarbital, henobarbital, secobarbital |
| **Anticoagulants** | bemiparin, certoparin, dalteparin, enoxaparin, nadroparin, parnaparin, reviparin, tinzaparin, unfractionated heparin, fondaparinux, rivaroxaban, dabigatran, apixaban, warfarin, coumadin |
| **Antiplatelets** | abciximab, anagrelide hydrochloride, aspirin/dipyridamole, cilostazol, clopidogrel hydrogen sulfate, dipyridamole, eptifibatide, prasugrel hydrochloride, ticagrelor, ticlopidine hydrochloride, tirofiban hydrochloride |
| **NSAIDs** | aspirin alone or in combination, salicylates alone or in combination bromfenac sodium, meclofenamate sodium, tolmetin sodium, celecoxib, diclofenac potassium or sodium, diclofenac sodium/misoprostol, diflunisal, etodolac, fenoprofen calcium, flurbiprofen, ibuprofen, ibuprofen/pseudoephedrine hydrochloride, indomethacin, ketoprofen, ketorolactromethamine, mefenamic acid, meloxicam, nabumetone, naproxen, naproxen sodium alone or in combination, oxaprozin, phenylbutazone, piroxicam, salsalate, sulindac, valdecoxib, tiaprofenic acid, fenbufen, tenoxicam |
| **Antihistamines** | hydroxyzine, promethazine, diphenhydramine, cetirizine, levocetirizine, desloratadine, fexofenadine, ebastine, loratadine, clemastine, chlorphenamine, rupatadine, mizolastine, ketotifen |
| **Anticholinergics** | atropine, benztropine, biperiden, doxazosin, flavoxate, glycopyrrolate, ipratropium, orphenadrine, oxitropium, oxybutynin, tolterodine, tiotropium, trihexyphenidyl, propiverine, solifenacin |
| **Antidiabetics** | **Insulin:** regular insulin, insulin lispro, insulin aspart, insulin glulisine, insulin zinc, isophane insulin neutral protamine hagedorn (nph), insulin glargine, insulin detemir  **Biguanide:** metformin, glipizide/metformin, glyburide/metformin, pioglitazone/metformin, rosiglitazone/metformin, repaglinide/metformin, sitagliptin/metformin, saxagliptin/metformin  **Dipeptidyl peptidase-4 Inhibitors:** sitagliptin, saxagliptin, linagliptin, sitagliptin/metformin, saxagliptin/metformin  **Glucagon-Like Peptide-1 Agonists**: abliglutide, dulaglutide, exenatide, liraglutide, lixisenatide  **Meglitinide Derivatives:** nateglinide, repaglinide, repaglinide/metformin  **Sodium-Glucose Co-Trasporter 2 Inhibitors**: canagliflozin, dapagliflozin, empagliflozin  **Sulfonylureas:** acetohexamide, chlorpropamide, glimepiride, glipizide, glyburide, tolazamide, tolbutamide, glipizide/metformin, glyburide/metformin, glimepiride/pioglitazone, glimepiride/rosiglitazone  **Thiazolidinediones:**pioglitazone, rosiglitazone, glimepiride/pioglitazone, glimepiride/rosiglitazone, pioglitazone/metformin, rosiglitazone/metformin  **α-Glucosidase Inhibitors:** acarbose, miglitol  Inpramlintide, glucagon-like peptide-1 receptor agonists |
| **Antihypertensives** | **Beta Blockers (BB):** carteolol, nadolol, nebivolol, penbutolol, pindolol, propranolol, sotalol, timolol, acebutolol, atenolol, betaxolol, bisoprolol, esmolol, metoprolol, carvedilol, labetalol  **Calcium Channel Blockers (CCB)**: bepridil, diltiazem, mibefradil, verapamil, amlodipine, clevidipine, felodipine, isradipine, nicardipine, nifedipine, nimodipine, nisoldipine  **Agents Affecting the Renin-Angiotensin-Aldosterone System**: benazepril, captopril, enalapril/enalaprilat, fosinopril, lisinopril, moexipril, perindopril, quinapril, ramipril, trandolapril, candesartan, eprosartan, irbesartan, losartan, olmesartan, telmisartan, valsartan, azilsartan, eplerenone, aliskiren  **Adrenolytics**: clonidine, guanabenz, guanfacine, methyldopa, guanadrel, guanethidine  **Alpha Blockers:** doxazosin, prazosin, terazosin  **Reserpine:** deserpidine, rauwolfia, reserpine  **Vasodilators:** cyclandelate, diazoxide, epoprostenol, ethaverine, fenoldopam, hydralazine, minoxidil, nylidrin, papaverine, tolazoline, treprostinil, trimethaphan  **Diuretics:** bendroflumethiazide, benzthiazide, chlorothiazide, chlorthalidone, hctz, hydroflumethiazide, indapamide, methyclothiazide, metolazone, polythiazide, trichlormethiazide, bumetanide, ethacrynic acid, furosemide, torsemide, amiloride, spironolactone, triamterene  **Fixed-Dose Combination Antihypertensive Agents:** atenolol/chlorthalidone, bisoprolol/hctz , metoprolol/hctz, nadolol/bendroflumethiazide, propranolol/hctz, timolol/hctz , benazapril/amlodipine, enalapril/diltiazem, enalapril/felodipine, trandolapril/verapamil, benazepril/hctz, captopril/hctz, enalapril/hctz , fosinopril/hctz, lisinopril/hctz, moexipril/hctz, quinapril/hctz, aliskiren/hctz, aliskiren/valsartan, olmesartan/amlodipine, telmisartan/amlodipine, valsartan/amlodipine, candesartan/hctz, eprosartan/hctz, irbesartan/hctz, losartan/hctz, olmesartan/hctz, telmisartan/hctz, valsartan/hctz, azilsartan/chlorthalidone, olmesartan/amlodipine/hctz, valsartan/amlodipine/hctz, amiloride/hctz, spironolactone/hctz, triamterene/hctz, prazosin/polythiazide, clonidine/chlorthalidone, guanethidine/hctz, methyldopa/chlorothiazide, methyldopa/hctz, deserpidine/hctz, deserpidine/methyclothiazide, rauwolfia/bendroflumethiazide, reserpine/chlorothiazide, reserpine/chlorthalidone, reserpine/hctz, reserpine/hydroflumethiazide, reserpine/methyclothiazide, reserpine/polythiazide, reserpine/trichlormethiazide, reserpine/hydralazine/hctz, hydralazine/hctz, hydralazine/isosorbide dinitrate |
| **Anti-Epileptics** | carbamazepine, ethotoin, fosphenytoin, lacosamide, lamotrigine, oxcarbazepine, rufinamide, phenytoin, clonazepam, diazepam, gabapentin, phenobarbital, pregabalin, primidone, tiagabine, ethosuximide, lorazepam, clobazam, vigabatrin, levetiracetam, ezogabine, divalproex sodium, felbamate, topiramate, valproate sodium, valproic acid and zonisamide, |
| **Anti-Emetic Neuroleptics** | metoclopramide, ondansetron, granisetron, droperidol, palonosetron  aprepitant, fosaprepitant |
| **Antidementia Drugs** | donepezil, rivastigmine, memantine, galantamine |
| **Narcotics** | fentanyl, meperidine, tramadol, methadone, pentazocine, oxycodone, hydrocodone, codeine, morphine, oxymorphone, hydromorphone, levorphanol |
| **Hormones** | estradiol cypionate, estradiol valerate, estradiol, ethinyl estradiol, estradiol benzoate, estradiol acetate, estradiol micronized, estrone, estrogens conjugated/meprobam, estrogens conjugated, estriol, chlorotrianisene, quinestrol, dienestrol, estrogens esterified, estropipate, estrogens conj. synthetic a, estrogens conj. synthetic b, estriol micronized, ethinyl estradiol/noreth ac, norethind ac/ethinyl estradiol, estradiol/noreth ac, estradiol/norgestimate, estradiol/levonorgestrel, estradiol/drospirenone, estrogen con/m-progest acet |
| **Stimulants** | dexmethylphenidate, dextroamphetamine, diethylpropion, lisdexamfetamine dimesylate, methylphenidate, modafinil, armodafinil, pemoline, phendimetrazine, phentermine, amphetamine |
| **For Dyslipidemia** | cholestyramine, colestipol, colesevelam, atorvastatin, fluvastatin, lovastatin, pitavastatin, pravastatin, simvastatin, rosuvastatin, nicotinic acid, ezetimibe, fenofibrate, bezafibrate, ciprofibrate, gemfibrozil |
| **For Parkinson’s disease** | levodopa, carbidopa, benserazide, dopamine agonists (apomorphine hydrochloride, bromocriptine, rotigotine, pramipexole, ropinirole, piribedil, cabergoline, lisuride), selegiline, rasagiline |

*NSAID* non-steroidal anti-inflammatory drug
